# Exploitation of data from breeding programs supports rapid implementation of genomic selection for key agronomic traits in perennial ryegrass

**DOI:** 10.1007/s00122-018-3121-7

**Published:** 2018-06-02

**Authors:** Luke W. Pembleton, Courtney Inch, Rebecca C. Baillie, Michelle C. Drayton, Preeti Thakur, Yvonne O. Ogaji, German C. Spangenberg, John W. Forster, Hans D. Daetwyler, Noel O. I. Cogan

**Affiliations:** 1 Agriculture Victoria Research, AgriBio, Centre for AgriBioscience, 5 Ring Road, Bundoora, VIC 3083 Australia; 2 New Zealand Agriseeds, 2547 Old West Coast Road, Christchurch, 7671 New Zealand; 3 School of Applied Systems Biology, La Trobe University, Bundoora, VIC 3086 Australia

## Abstract

**Key message:**

Exploitation of data from a ryegrass breeding program has enabled rapid development and implementation of genomic selection for sward-based biomass yield with a twofold-to-threefold increase in genetic gain.

**Abstract:**

Genomic selection, which uses genome-wide sequence polymorphism data and quantitative genetics techniques to predict plant performance, has large potential for the improvement in pasture plants. Major factors influencing the accuracy of genomic selection include the size of reference populations, trait heritability values and the genetic diversity of breeding populations. Global diversity of the important forage species perennial ryegrass is high and so would require a large reference population in order to achieve moderate accuracies of genomic selection. However, diversity of germplasm within a breeding program is likely to be lower. In addition, de novo construction and characterisation of reference populations are a logistically complex process. Consequently, historical phenotypic records for seasonal biomass yield and heading date over a 18-year period within a commercial perennial ryegrass breeding program have been accessed, and target populations have been characterised with a high-density transcriptome-based genotyping-by-sequencing assay. Ability to predict observed phenotypic performance in each successive year was assessed by using all synthetic populations from previous years as a reference population. Moderate and high accuracies were achieved for the two traits, respectively, consistent with broad-sense heritability values. The present study represents the first demonstration and validation of genomic selection for seasonal biomass yield within a diverse commercial breeding program across multiple years. These results, supported by previous simulation studies, demonstrate the ability to predict sward-based phenotypic performance early in the process of individual plant selection, so shortening the breeding cycle, increasing the rate of genetic gain and allowing rapid adoption in ryegrass improvement programs.

**Electronic supplementary material:**

The online version of this article (10.1007/s00122-018-3121-7) contains supplementary material, which is available to authorized users.

## Introduction

Perennial ryegrass (*Lolium perenne* L.) is the most important temperate pasture species on a global basis and plays a dominant role as the primary feed-base in dairy systems in northern Europe, Australia, New Zealand and other regions. Ryegrass displays the desirable characteristics of high forage yield and nutritive quality and superior tolerance to grazing. However, only limited genetic gain has been obtained over the past century, with estimates of 0.25–0.6% annual genetic improvement for dry matter production (Wilkins and Humphreys 2003; Woodfield 1999). Several aspects of ryegrass biology have contributed to this problem, such as an obligate outbreeding reproductive habit and associated high levels of genetic diversity, which have limited the fixation of desirable gene variants; a prevalence of target agronomic traits under complex genetic control with substantial environmental modification (and so typically of low-to-moderate heritability); and shortcomings of current phenotypic assessment methodology. Ryegrasses are cultivated as genetically heterogeneous populations in a pasture sward, and agronomic performance is evaluated on a sward-specific basis. However, such assessment is not appropriate for individual plants that are the target of selection in the early stages of breeding programs, which are typically grown under spaced or semi-spaced conditions in order to identify elite genotypes as parents for synthetic varietal production. Moreover, limited correlation has been observed between biomass yield estimates from spaced plants and corresponding sward-based performance (Wang et al. 2016a, b).

Significant incremental development of genomic resources for perennial ryegrass, such as expressed sequence tags (ESTs), genome survey sequences, transcriptome collections and most recently, whole-genome sequences (Sawbridge et al. 2003; Farrell et al. 2014; Byrne et al. 2015) has permitted the development of sequence-tagged molecular genetic marker systems such as simple sequence repeats (SSRs) and single nucleotide polymorphisms (SNPs) (Forster et al. 2008). These and other marker systems have been used for trait-dissection studies, largely based on biparental mapping populations (Shinozuka et al. 2012), in concert with increasingly detailed phenotypic measurements. However, individual marker-trait associations are of highly restricted value for application in breeding programs, due to the genetic complexity of traits and heterogeneous nature of populations. In contrast, genomic selection, which exploits a large number of genome-wide-distributed sequence polymorphisms in linkage disequilibrium with causal gene variants, has high potential for implementation in the breeding of species such as perennial ryegrass (Hayes et al. 2013). The value of such an approach has been verified by comprehensive simulation analysis of a current commercial breeding program (Lin et al. 2016).

Genomic selection allows prediction of the phenotypic performance of selection candidates based only on genotype data, after marker effects are estimated in a reference population that has been subjected to both genotypic and phenotypic evaluation (Meuwissen et al. 2001). In the context of ryegrass breeding, this may permit prediction of the in-sward performance of individual plants (as selection candidates) from a reference population of genotyped populations/varieties grown as swards. As such a process has not previously been feasible, dramatic improvements in selection accuracy and genetic gain within breeding programs are expected (Lin et al. 2016). In addition, selection candidates do not require phenotypic assessment in order to estimate genetic potential, which is currently performed over a period of several years in pasture plant breeding programs. Genomic breeding values can hence be calculated at the seedling stage, which greatly reduces the generation interval and consequently increases genetic gain. However, a number of factors that influence the accuracy of genomic selection, such as reference population size, trait heritability and genetic diversity of breeding populations must be considered.

The global genetic diversity of perennial ryegrass is large (Pembleton et al. 2016) and so would require an extensive reference population. Diversity within a single breeding program should be lower, due both to sub-selection of founder genotypes from a broader range of germplasm, and the cyclic nature of many programs, such that individuals from pre-existing elite varieties are often chosen as parents for a next round of top-crosses. As a consequence, the genetic relationship between selection candidates and breeding program-specific reference populations would be expected to increase over time. Active breeding programs cumulatively generate extensive phenotypic data covering the genetic and phenotypic diversity of their germplasm, but this information has historically been used in isolation within each year or generation to select the best performing plants or populations. However, the data also represent a potential resource for the development of breeding program-specific reference populations for genomic selection. Genotypic data obtained from retained biological materials, such as leaf tissue or seeds, can be combined with the phenotypic data to establish a reference population from which marker effects can be estimated. This approach also offers a significant advantage in terms of timely implementation, as de novo construction and characterisation of reference populations is a logistically complex and time-consuming process.

Although a number of studies that describe genomic prediction accuracies in ryegrass have recently been published, none have demonstrated the ability to predict seasonal biomass yield consistently across a series of seasons and years. Fè et al. (2016) explored genomic selection for seed production related traits, forage quality and crown rust resistance in commercial germplasm achieving moderate correlations between average phenotypes and GEBVs in the range of c. 0.2–0.56. Fè et al. (2015) considered the trait of heading date, and using a cross-validation scheme achieved correlations between average phenotype and GEBVs ranging from 0.52 to 0.9. Grinberg et al. (2016) used data from previous generations (containing parental genotypes) to predict the performance of derived half-sib populations using genomic best linear unbiased prediction (GBLUP) and machine learning models. Although prediction accuracies for nutrient quality components (such as water-soluble carbohydrate content) were moderate (0.454–0.598), those for more complex traits such as biomass yield, which was predicted only as total (not seasonal) yield, ranged from − 0.013 to 0.275, and was highly variable between different training populations and years.

Recently, Lin et al. (2016) simulated implementation of genomic selection within a commercial ryegrass breeding program (that of New Zealand Agriseeds [NZA]) to estimate the levels of prediction accuracy and resulting genetic gain for key breeding targets such as biomass yield and persistency. Prediction accuracies of 0.17 and 0.19 for sward/plot-based persistency and yield, respectively, were obtained, while a higher value (0.7) was obtained for the simpler trait of flowering time. Based on these results, the genetic gain expected from genomic selection was calculated to be up to sixfold greater than for standard phenotypic selection. Phenotypic data are available from the same program, extending over 15 years of breeder’s plot trials, in addition to retained seeds from each derived synthetic population. The present study was therefore based on a combination of genotypic data from pooled samples of each population with the historical phenotypic data to provide genomic predictions of performance in successive cycles. This process has demonstrated the potential for exploiting such data for rapid implementation of genomic selection and achieving moderate-to-high prediction accuracies for key agronomic traits.

## Materials and methods

### Phenotypic data

Phenotypic data from sward-based trials of diploid ryegrass synthetic populations for yield and heading date were recorded for the period from 1997 to 2014. Two trials for biomass yield were sown annually and phenotypically assessed over a 2-year period at the premises of NZA at Courtenay, Christchurch, New Zealand. Each trial entailed assessment of 20–25 synthetic populations, sown as 6 m^2^ plots, with threefold replication, for a total of 772 synthetic populations over the 18-year period. Trials were structured in order to simulate on-farm management conditions, yield cuts being generally performed every 1–2 months, when plants reached the three-leaf stage of growth. Three broadly adopted commercial cultivars (Bronsyn, Alto and Trojan) were commonly used as reference varieties across each trial and year, either individually or in combination. Trials from 1997 to 2008, 2006 to 2011 and 2011 to 2014 used Bronsyn, Alto and Trojan, respectively.

Yield data were analysed as a two-stage process using residual maximum likelihood (REML) methods implemented with the software package ASReml v3 (Gilmour et al. 2009). Each cut for yield (across the 2 years) was allocated to one of 5 seasonal periods, as defined by the New Zealand forage value index for the South Island (Chapman et al. 2012). These were autumn (March, April and May), winter (June and July), early spring (August and September), late spring (October and November), and summer (December, January and February), based on the period with the highest number of growth days, along with average yield across all periods. Yield trials were analysed in a randomised incomplete block design for each period, with block fitted as a random effect to adjust for spatial variation and synthetic population as a fixed effect. The best linear unbiased estimate (BLUE) of seasonal phenotypic performance for each population was accompanied with a weight (using the quantifier !TWOSTAGEWEIGHTS), which is a function of replication and error variance and represents the degree of uncertainty of the BLUEs (Smith et al. 2001). Following individual within-trial analyses, BLUEs for the 1997–2008 interval were scaled, within each trial, relative to the BLUE value for Bronsyn set as zero. Best linear unbiased predictors (BLUPs) for 1997–2008, including the weights from the previous BLUE analysis, were then calculated, and subsequently the difference in performance between Bronsyn and Alto was calculated and used to scale all trials from 2006 to 2011 relative to the BLUE value for Bronsyn set as zero. This process was repeated for the 2006–2011 interval in order to calculate the difference in performance between Trojan and Bronsyn, permitting scaling relative to Bronsyn for 2011–2014. All scaled BLUEs from 1997 to 2014 were then combined into a single BLUP analysis (for each season), including the weights, to calculate final predictions of the performance for each synthetic population relative to Bronsyn, here on termed adjusted phenotypic values.

Phenotypic data from heading date trials spanned the interval from 2002 to 2012, from a smaller set of synthetic populations, the majority of which overlapped with those used in the yield trials. Trials for heading date were sown as 1 m rows, with threefold replication. Heading date was defined as number of days elapsed since 1st September at which 50% of plants within the synthetic population were in flower. Similar to the biomass yield trial analysis, a two-stage process was performed for the heading date trials, with Bronsyn as a common reference cultivar from 2002 to 2008, and cultivar Bealey from 2004 to 2012, here on termed adjusted phenotypic values.

### Genotypic data

Seed batches from 714 of the 861 trialled synthetic populations (772 from yield trials, 568 from heading date trials) were available for genotyping. Synthetic populations were typically derived from 4 to 6 parent synthetic crosses over an 18-year breeding period. Seeds from each population were germinated on filter paper. A single leaf was harvested from each of sixty germinants and used for pooled RNA extraction using RNAeasy (QIAGEN, Hilden, Germany) following manufacturer’s instructions. Purified RNA was then processed for genotyping-by-sequencing as described by Malmberg et al. (2017). RNA-Seq libraries were sequenced on a HiSeq 2000 or 3000 platform (Illumina, San Diego, California, USA) to generate approximately 60 million reads (30 million paired) per synthetic population. Sequence reads were aligned to the perennial ryegrass transcriptome assembly described by Shinozuka et al. (2017) using *BWA*-*mem* (Li and Durbin 2009), and a total of 127,000 variants were called using *samtools mpileup v1.3.1* (Li et al. 2009) and *bcftools v1.3.1*. Allele frequency of the reference nucleotide at variant sites for each sample was calculated with in-house scripting using the allele Depth (AD) field in the resulting vcf file. Loci for which more than 50% of the synthetic populations displayed fewer than 100 reads were deemed unreliable and were removed. Subsequently, SNPs predicted from less than 40 reads were re-coded as missing data, which was then imputed using custom in-house scripts, based on the method of linkage disequilibrium k-nearest neighbours imputation (LD-kNNi), as described by Money et al. (2015) which was adapted in-house to impute allele frequencies rather than bi-allelic genotypic states. Parameters for imputation were 11 nearest neighbours (k) and 17 closest (LD) loci.

### Genomic prediction

Genomic prediction accuracy was explored by calculating GEBVs for all synthetic populations within each year, using only those that were trialled in previous years as the reference population, henceforth described as forward genomic prediction. Yield in the first and second year of production was treated as the same trait. Accuracy was calculated as the correlation between predicted GEBVs and observed phenotypes without further adjustment. For biomass yield, as the field trials were assessed for 2 years, the synthetic populations sown in the previous year to that of those being predicted were excluded from the reference population, in order to ensure a conservative estimate of prediction accuracy. Additionally, the effects of separation periods between comparator populations of 2 and 3 years were assessed. To reduce any bias arising from large differences in reference population size, the additional gaps of 2 and 3 years were only compared for accuracy of prediction from 2004 onwards. At each iteration of the reference population, when cycling across prediction years, SNP loci were filtered to retain only those where the variance across samples (in the reference population) for the called allele frequencies was > 0.01. After filtering, a genomic relationship matrix was calculated following the method of Yang et al. (2010), where the bi-allelic genotypic classes (0, 1, 2) were replaced with twice the allele frequency of the reference allele (i.e., 100% reference was re-coded as ‘2’). Therefore, genotypes could take on a real number ranging from 0 to 2. As two distinct genetic backgrounds within the breeding program were known to exist from consideration of pedigree data (data not shown), cluster analysis of genomic relationships through partitioning around medoids with the *pamk* function in the R package *fpc* (Hennig 2015) was implemented to genetically define the two main clusters. Cycling across each year, cluster analysis was re-performed on only the respective reference and prediction synthetic populations (independent of the whole dataset). The cluster with the most samples originally assigned to the original main cluster 1 was labelled as Group A, and similarly the cluster with the most samples originally assigned to main cluster 2 was labelled Group B. Any additional smaller clusters were labelled as ‘unassigned’ and were excluded from genomic prediction. If the number of samples in the group specific reference population was less than 50, the group was excluded from genomic prediction assessment. The groups were then independently assessed for genomic prediction accuracy, as well as in combination (global). Genomic predictions were calculated using the BayesA model as proposed by Meuwissen et al. (2001) and implemented using the R package BGLR (Pérez and de los Campos 2014);

$$\varvec{y} = u1_{n} + X\varvec{v} + \varvec{e}$$

where *y* is the trait of interest (i.e., BLUPs for biomass or heading date of the respective synthetic varieties), *u* is the population trait mean, $1_{n}$ is a vector of ones, n, the number of records, *X* is a matrix of genome-wide distributed SNP makers coded as the reference allele frequency, ***v*** is a vector of random SNP effects estimated from the reference population where each SNP effect is $\varvec{v}_{i} \sim N( {0, \sigma_{{v_{i} }}^{2} })$, and $\varvec{e} \sim N( {0, \sigma_{e}^{2} })$ is a vector of residual errors. The variance of each SNP *i*, $\sigma_{{v_{i} }}^{2}$ was sampled from an inverted Chi-square distribution using the default degrees of freedom and the scaling parameter determined by BGLR from a trait heritability calculated as the mean of the within-trial heritability of the reference population for the relevant trait. The error variance $\sigma_{e}^{2}$ was also sampled from an inverted Chi-square distribution with default BGLR parameters.

Fitting of BayesA genomic prediction models and estimation of marker effects were performed with 12,000 iterations, discarding the first 2000 as burn-in. Genomic prediction accuracy was calculated as the correlation between adjusted phenotypic values and predicted GEBVs. To mitigate the error in the correlation resulting from poor data distribution due to the small numbers of predicted GEBVs in some years/groups, the correlation was bootstrapped. Bootstrapping was performed for each season and year by sampling from the predicted GEBVs and corresponding adjusted phenotypic values, with replacement, the same number of complete records and recalculating the correlation. This bootstrapping process was repeated 10,000 times. Adjusted phenotypic values were also regressed on all predicted GEBVs, for each season, to calculate the bias in genomic prediction, represented by the slope coefficient. The ideal slope coefficient of 1 represents no bias in the prediction of GEBVs, while slope coefficients greater than one represent under-prediction.

## Results

### Phenotypic data and heritability

The number of biomass-specific harvests per seasonal period (across the 2 years) ranged from 1 to 5, 1 to 3, 1 to 4, 3 to 7 and 2 to 7 for autumn, winter, early spring, late spring and summer, respectively. Across all trials and seasons, phenotypic values adjusted for spatial effects were calculated. Heritability estimates for biomass yield were calculated based on the variance components computed from the within-trial REML analyses and were highly variable between trials, years and seasons, ranging from 0.05 to 0.81, with a mean of 0.42 (Online Resource 1).

Winter, which on average had the least number (1) of harvests within each year, was equal with early spring (1.2 harvests/year) for the highest mean heritability value of 0.55, followed by autumn, late spring and summer, with values of 0.43, 0.41 and 0.26, respectively. In contrast, the heritability of heading date was consistently high, varying between 0.65 and 0.93 with a mean of 0.86.

### Genotype data

On average 55 million sequencing reads were obtained for each synthetic population, with a mean mapping percentage of 91%. Removal of SNP loci with more than 50% missing data obtained a remaining set of 54,569, for which the mean missing data level was 12%. Missing data were imputed with an expected accuracy (calculated as the correlation between true and imputed genotype) of 90%, based on simulation of additional missing data (data not shown). Further filtering for variation in both the training and prediction populations in 1999 resulted in 28,573 segregating polymorphisms for genomic prediction, increasing across the years to 29,766 in 2014, likely due to novel genetic variation as new synthetic populations were added to the reference population.

### Clustering of genetic backgrounds

**Fig. 1:**
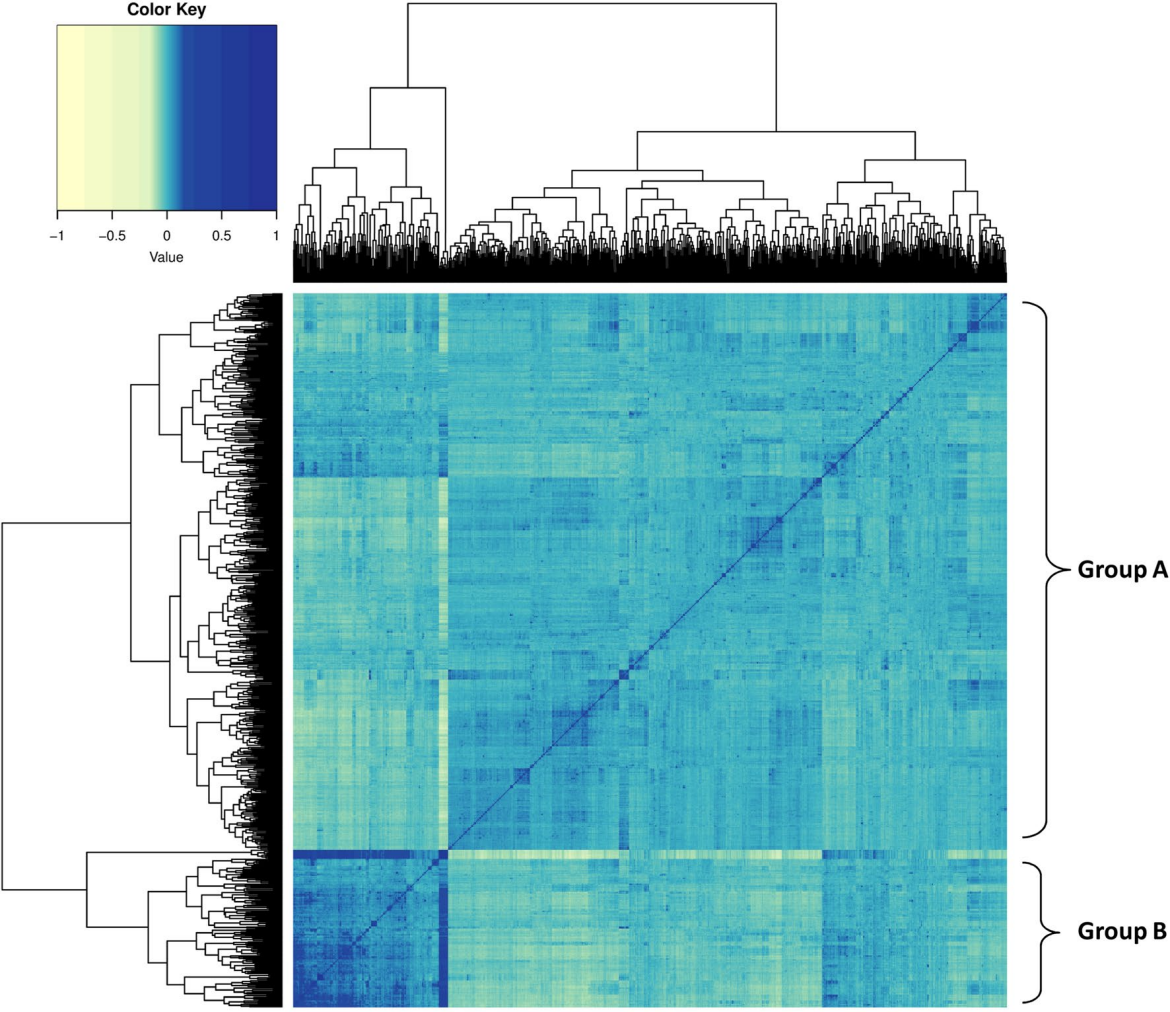
Heatmap of genomic relationships illustrating the distinct clustering of two genetic backgrounds, Group A and Group B

**Fig. 2:**
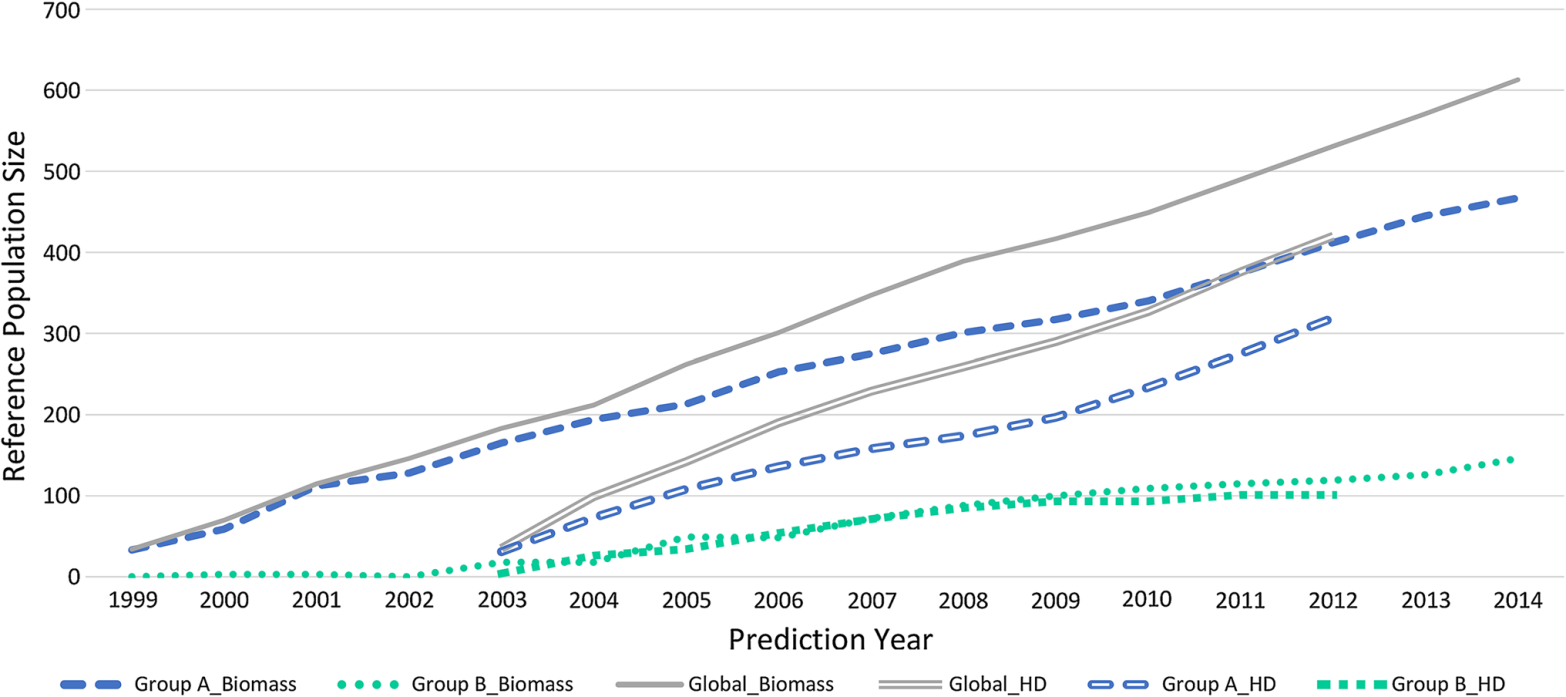
Reference population size across each prediction year for global biomass (grey solid line), Group A biomass (blue solid dashed), Group B biomass (green round dotted), global heading date (grey double line), Group A heading date (blue hollow dashed), Group B heading date (green square dotted)

Clustering of synthetic populations based on genomic relationship identified two distinct genetic backgrounds, designated groups A and B (Fig. 1). Group A contained 475 (78%) of the 714 genotyped samples, of which 467 and 299 had biomass yield and heading date data, respectively. Group B was represented by 239 synthetic populations (22%) of which 233 and 163 had biomass yield and heading date data, respectively. The proportion of predicted synthetic populations in each group varied according to year. However, a general trend of increased inclusion of Group B individuals over time was observed, from a minimal level prior to 2005 (Fig. 2).

### Accuracy of genomic predictions for biomass yield

#### Global accuracy across groups

The average forward genomic prediction accuracy for mean annual biomass yield across both groups A and B (in the absence of clustering, referred to as ‘global’) was 0.310 (Table 1). For individual seasons, the average prediction accuracy ranged from 0.205 in late spring to 0.589 in early spring. Although summer displayed the largest standard deviation across years, late spring was the most variable seasonal period relative to prediction accuracy, while early spring was the least variable (Table 1).

**Table 1:** Global forward genomic prediction accuracy for biomass across years and seasons

Year	Global prediction					
	Average	AUT	WIN	ESP	LSP	SUM
1999	0.264	0.287	0.154	0.791	0.018	0.131
2000	0.259	0.221	0.351	0.310	− 0.114	0.265
2001	0.363	0.357	0.276	0.636	− 0.016	0.590
2002	0.616	0.557	0.374	0.790	0.052	0.446
2003	0.282	0.359	0.299	0.737	0.158	0.395
2004	0.246	0.266	0.292	0.588	0.466	0.431
2005	0.035	0.211	0.018	0.582	0.273	− 0.104
2006	0.159	0.118	0.167	0.011	0.220	0.280
2007	0.352	0.485	0.292	0.576	0.149	0.374
2008	0.214	− 0.007	0.425	0.726	0.108	0.003
2009	0.363	0.416	0.200	0.492	0.532	0.309
2010	0.575	0.339	0.114	0.659	0.206	0.405
2011	0.159	− 0.049	0.053	0.667	0.515	0.472
2012	0.423	0.051	0.054	0.682	0.137	− 0.071
2013	0.496	− 0.100	0.271	0.514	0.346	0.381
2014	0.155	0.259	0.270	0.661	0.222	0.178
Mean	0.310	0.236	0.226	0.589	0.205	0.280
SD	0.160	0.190	0.122	0.196	0.187	0.202

#### Accuracy within groups

**Fig. 3:**
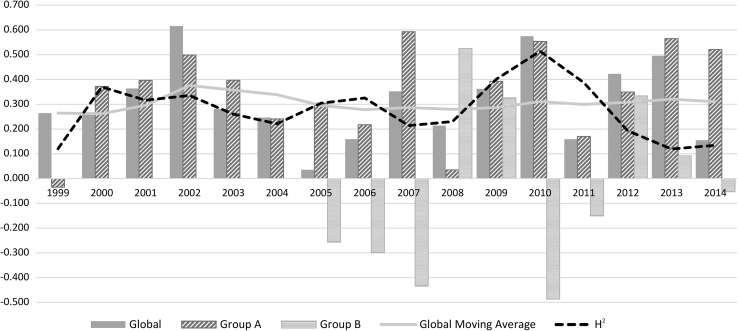
Individual year global (grey solid bars) Group A (blue diagonal stripe bars) and Group B (green horizontal stripe bars) average seasonal biomass accuracy. Moving global average and heritability across years is represented by the yellow (solid) and black (dashed) lines, respectively

Clustering based on genomic relationships, and consequent prediction independently within the two groups, resulted in an accuracy of forward genomic predictions for average annual biomass yield of 0.348 and − 0.041 in Groups A and B, respectively (Table 2). Additionally, the accuracy was higher for each individual period (apart from early spring), when only using Group A synthetic populations (Table 2). The average accuracy in Group A by 2014 for autumn, winter, early spring, late spring and summer was 0.348, 0.255, 0.577, 0.240 and 0.297, respectively. Across most years clustering of Group A provided improvements in average seasonal prediction accuracy over global prediction (Fig. 3). Those years where no improvement was observed were typically characterised by already moderate global prediction accuracies (Fig. 3). Group B had highly variable and low prediction accuracy across years (Fig. 3). The mean seasonal accuracy for autumn, winter, early spring, late spring and summer was − 0.052, − 0.028, 0.208, 0.119 and 0.007, respectively. Apart from average (1.03) measurements, minor bias in the prediction of GEBVs in Group A was observed in autumn (1.11), moderate bias in early spring (1.19) and late spring (1.24), with values being under-predicted, while summer (0.87) and winter (0.62) showed an over-prediction bias. Global prediction had minor over-prediction bias in average (0.94) and late spring (0.90), moderate over-prediction bias in autumn (0.76), summer (0.74) and winter (0.533) while early spring (1.11) had minor under-prediction bias.

**Table 2:** Groups A and B forward prediction accuracy for biomass across seasons and years

Year	Group A prediction						Group B prediction					
	Average	AUT	WIN	ESP	LSP	SUM	Average	AUT	WIN	ESP	LSP	SUM
1999	− 0.035	0.547	0.312	0.207	− 0.051	− 0.032						
2000	0.372	0.268	0.482	0.376	− 0.217	0.298						
2001	0.396	0.550	0.213	0.636	− 0.119	0.439						
2002	0.498	0.584	0.259	0.647	0.096	0.655						
2003	0.397	0.555	0.463	0.748	0.336	0.512						
2004	0.241	0.327	0.153	0.655	0.589	0.376						
2005	0.304	0.317	0.128	0.684	0.329	− 0.017	− 0.257	0.392	0.025	0.431	0.050	− 0.292
2006	0.218	0.530	0.355	0.109	0.296	0.548	− 0.299	− 0.405	− 0.150	0.215	− 0.254	− 0.112
2007	0.592	0.631	0.598	0.806	0.345	0.318	− 0.435	− 0.186	− 0.483	0.176	− 0.300	− 0.161
2008	0.035	0.201	0.424	0.759	0.250	− 0.060	0.524	− 0.524	0.280	0.368	0.030	− 0.310
2009	0.393	0.314	0.269	0.534	0.392	0.371	0.325	0.596	− 0.572	− 0.275	0.639	0.978
2010	0.554	0.390	− 0.026	0.646	0.164	0.402	− 0.486	− 0.974	0.322	− 0.402	0.233	− 0.901
2011	0.170	− 0.036	− 0.008	0.651	0.461	0.300	− 0.151	0.317	− 0.077	0.083	0.387	0.249
2012	0.349	0.200	0.125	0.594	0.058	− 0.041	0.334	0.207	− 0.011	0.452	0.341	0.219
2013	0.565	− 0.159	0.139	0.456	0.438	0.234	0.091	− 0.302	0.278	0.364	0.381	0.188
2014	0.521	0.513	0.197	0.718	0.469	0.442	− 0.053	0.359	0.104	0.673	− 0.314	0.216
Mean	0.348	0.358	0.255	0.577	0.240	0.297	− 0.041	− 0.052	− 0.028	0.208	0.119	0.007
SD	0.185	0.227	0.176	0.197	0.231	0.224	0.348	0.502	0.308	0.333	0.331	0.492

In addition to the standard 1-year gap between reference and prediction populations, gaps of 2 or 3 years were also assessed (from 2004 onwards) to assess the impact on prediction accuracy when the reference population becomes further distanced from the selection candidates (Online Resource 2). For a 2-year gap annual average biomass and the equivalent values for all of the seasons, remained relatively (< 0.05 unit change) unaffected for both global and Group A prediction. When a 3-year gap was evaluated, all values for global prediction remained relatively unaffected, apart from later spring (reduction of 0.05), while Group A prediction had reductions in early spring, late spring and summer, of 0.07, 0.10 and 0.06, respectively.

### Accuracy of genomic predictions for heading date

The genomic prediction accuracy across years for heading date was consistently high in the absence of grouping, achieving a rolling average of 0.76 by 2012 (Table 3). The lowest level of accuracy (0.69) was observed when predicting the 2009 trial. Clustering of samples into Group A provided no significant benefit to heading date prediction accuracy, as average accuracy only increased to 0.78 when Group A alone was used. However, a reduction to 0.661 in average prediction accuracy for Group B was observed. Minimal prediction bias was observed for heading date, for both global and Group A prediction with a slope coefficient of 1.16, while under-prediction bias was observed in Group B (1.92).

**Table 3:** Forward genomic prediction accuracy for heading date across years

Year	Global prediction		Group A prediction		Group B prediction	
	Accuracy	Moving average	Accuracy	Moving average	Accuracy	Moving average
2003	0.806	0.806	0.776	0.776		
2004	0.741	0.774	0.809	0.793	0.325	0.325
2005	0.800	0.783	0.886	0.824	0.524	0.425
2006	0.746	0.773	0.732	0.801	0.225	0.358
2007	0.802	0.779	0.815	0.804	0.519	0.398
2008	0.792	0.781	0.810	0.805	0.797	0.478
2009	0.692	0.769	0.776	0.801	1.000	0.565
2010	0.713	0.762	0.747	0.794	0.964	0.622
2011	0.742	0.759	0.651	0.778	0.950	0.663
2012	0.780	0.761	0.804	0.781	0.643	0.661
Mean	0.761		0.781		0.661	
SD	0.040		0.062		0.285	

Moving average is calculated as the average of current and all prior years

## Discussion

### Factors affecting accuracy of genotypic analysis

This study has demonstrated the ability to efficiently genotype ryegrass synthetic populations (which are composed of distinct individuals that are heterozygous at multiple genomic loci) using the transcriptome-based genotyping-by-sequencing method of Malmberg et al. (2017). A number of studies have employed different depths of sampling within populations to accurately represent the genetic diversity within a variety/population and relationships between. Most recently, Pembleton et al. (2016) demonstrated that 48 or more individual samples per ryegrass variety/population are required for such studies. As a consequence, a larger number (60 or more germinants per synthetic population) were harvested and subjected to pooled RNA extraction in the present study. For each synthetic population, allele frequency information for c. 127,000 SNP loci was generated, of which c. 55,000 loci were reliably called with sufficient depth across the majority of synthetic populations. Of these, c. 46% were found to be sufficiently polymorphic across synthetic populations to be informative in genomic selection models.

### Effects of population structure on accuracy of genomic prediction

Both population structure and distant genomic relationships between members of reference populations and selection candidates have the potential to negatively impact on genomic prediction accuracies. Two sub-populations or groups were identified within germplasm of the commercial breeding program, of which the first (group A) accounted for c. 67% of the overall dataset. Although Group B, corresponding to accessions of European origin, was generally only a minor component of the trialled varieties, the degree of differentiation between the two groups was sufficient enough to reduce prediction accuracies for biomass in later years when Group B varieties were more commonly used. Removal of Group B from the dataset generally resulted in an improvement in accuracy for Group A and in some seasons reduced variation between years. The reduced prediction accuracy is probably due to an insufficient reference population size for Group B, both in total and over successive years. This effect is likely to be alleviated in the future, as more Group B varieties are trialled and added to the reference population. This result, however, demonstrates the desirability of prior knowledge of genetic diversity and structure within breeding programs when applying genomic selection, which when absent may lead to poor prediction accuracies, incorrect selection decisions and consequent reduced genetic gain. Genetic clustering algorithms, as demonstrated in this study, will identify any such structure, and which varieties are likely to be vulnerable to such effects until adequate reference populations are developed. For highly diverse species such as perennial ryegrass, large commercial breeding programs will initially require reference populations that are specific to multiple genetic backgrounds. Depending on how such germplasm pools are maintained over time, and levels of introgression between pools, a single reference population may ultimately be consolidated and provide adequate accuracy across all genetic backgrounds.

### Accuracy of genomic prediction for biomass yield

Moderate levels of prediction accuracy were achieved for biomass yield in global and Group A varieties across all seasons for a majority of years, when using a BayesA model. A GBLUP genomic prediction model was also implemented, but revealed no significant differences when compared to the BayesA result (unpublished data). In general, average annual biomass was predicted with accuracy values above 0.2 (apart from for 2005, 2006, 2011 and 2014 for global, and 1999, 2008 and 2011 in group A), and a rolling average accuracy of 0.31 and 0.35 was obtained after 16 years of predictions for global and Group A, respectively. These values are similar to that reported by Annicchiarico et al. (2015) for cross-population biomass prediction accuracy in Lucerne, another outbreeding forage species. Moderate prediction bias was observed for Group A biomass yield, ranging from under-prediction in early spring and late spring to over-prediction in winter and summer. Although prediction bias was observed in some seasons, this would only have a detrimental impact on selection if plants with GEBVs were compared with plants with breeding values obtained from other methods or datasets, such as pedigree based values. However, this is not the proposed method for implementation of genomic selection in ryegrass, which is for breeding values of all synthetic populations to be genomically predicted, similar to that demonstrated (Lin et al. 2016). Prediction bias can also negatively impact selection accuracy if seasonal biomass predictions are combined in a across season selection; however, this can be accounted for by incorporating correction factors into a selection index.

As observed in previous studies (Muranty et al. 2015), prediction accuracies generally followed the order of heritability across seasons, with the highest accuracies (0.59, average) observed in early spring, and a lower accuracy (0.28, average) observed in summer. Although late spring assessments displayed relatively high heritability, this was the seasonal period that showed the second least accurate predictions. Ryegrass is known to exhibit an interaction effect between heading date and spring biomass yield traits (Sampoux et al. 2011), which may have affected the prediction accuracy. Although BayesA models were explored that included heading date as a fixed effect factor, no significant improvement in accuracy was observed (unpublished data). In the present study, whole biomass harvests were assigned to the period with most days of growth, although a significant proportion may have occurred in an adjacent period. The interaction effect, when combined with timing of previous harvest (which would vary across trials), would have at least partially determined which seasonal periods were allocated harvests that related to heading date-induced spring growth. Such variation may have contributed to the lower prediction accuracies characteristic of late spring measurements. New technologies for non-destructive phenotypic measurement of forage yield (such as normalised difference vegetation index) may provide a deeper understanding of mechanisms controlling spring biomass yield, so accurately assigning daily/weekly growth rates to specific seasons, rather than aggregating whole biomass harvests into the seasonal period with most days of growth.

Although average prediction accuracy values for each season were positive and moderate in magnitude, poor or negative values were observed for some years. It must be acknowledged that the reference population is constructed from phenotypic data across a series of years, which consequently represents a variety of temporally distinct seasonal environments with the possibility of G x E interactions. Consequently, the derived genomic equations and subsequent predictions are likely to represent average performance across various environments, and so correlations may have been reduced for years or seasons characterised by atypical environments. In these circumstances, the genomic predictions may still provide a true representation of performance in a standard year/environment. Phenotypic selection over years/seasons for which the environment was atypical may have led to reduced or even negative genetic gain.

The observation of moderate accuracy across seasons and years not only demonstrates the general ability to apply genomic selection to commercial perennial ryegrass breeding, but also potential for genomic selection of varieties with specific biomass growth profiles across seasons. Home-grown forage is the lowest cost feed source for dairy farms in both Australia and New Zealand, and utilisation of this resource provides much of the competitive ability of these nations on the international dairy market (Chapman et al. 2009). Close matching of the growth curve of pasture crops, such as ryegrass, to the feeding requirements of grazing dairy cattle can provide significant economic and production benefits to farmers, through reduced reliance on supplementary feeding over summer, autumn and winter, and diminished excess pasture production and related management costs over late spring (Chapman et al. 2009; de Klein 2001; Rawnsley et al. 2013; Stewart and Hayes 2011). Combination of the seasonal biomass prediction equations with recently developed economic values for seasonal biomass (Chapman et al. 2012) will enable the effective implementation of economic value selection indexes to assist in breeding and selection decisions.

In order to assess the optimal rate of update for the reference population (and consequently the prediction equation), in order to maintain moderate prediction accuracies, increased intervals of 2 and 3 years were compared to the standard 1-year gap. Although reduced accuracy was observed for both late spring and summer, the impact was generally limited across seasons, such that average seasonal biomass prediction accuracy remaining unchanged. This is probably due to the length of time that elapses between trialling of a synthetic population and synthesis of a new derived population. Although elite varieties will be recycled into the breeding program as new parents at various stages, at least 3–6 years will elapse before any derivative is trialled. Consequently, even with a 3-year gap, parental genotypes or closely related genotypes will be present within the reference population.

### Accuracy of genomic prediction for heading date

Prediction accuracy for heading date was consistently high across years, with a mean value of 0.76. Although effects of genetic background on biomass prediction accuracy were observed, much smaller effects were observed for heading date. Heading date is known to be under the control of a smaller subset of genes (Fè et al. 2015), as compared to biomass yield, which is assumed to be under complex control from a large number of genes, each of small individual magnitude. Due to the simpler genetic control of heading date, the ability to predict marker effects that explain this genetic variance across different germplasm pools and with smaller reference populations is enhanced. This is further supported by the minimal prediction bias observed for heading date, indicating that the magnitude of estimated marker effects closely matches the true effects. The BayesA model fitted in the present study assumes that each marker/gene has an effect on the phenotypic trait of interest, which does not accurately reflect the known genetic architecture of the heading date trait. Nonetheless, the BayesA model effectively predicted heading date with high accuracy, close to that expected based on an average heritability of 0.86. The ability of BayesA to effectively predict flowering date in other species has previously been reported (Li et al. 2015; Tayeh et al. 2015) and is probably due to the ability of the model to shrink marker effects towards zero if they do not explain components of phenotypic variance.

Phenotypic selection for traits of lower genetic complexity such as heading date and also awning, leaf width and length and growth habit, is currently required to achieve registration of Plant Breeders Rights/Plant Variety Rights, under distinctiveness, uniformity and stability (DUS) standards (Wang et al. 2016a, b). However, compared to biomass yield, these traits have minimal (if any) direct impact on agronomic performance and instead limit genetic gain by increasing the length of the breeding cycle (through a requirement for additional evaluation trials) and hindering direct agronomic selection (Wang et al. 2016a). Until systems are altered to allow registration based on genotypic profile, genomic selection (as demonstrated for heading date) may permit selection for discriminatory characteristics at the same time as more important agronomic traits, without requiring additional lengthy and laborious trials which impose negative impacts on genetic gain.

## Conclusions

The NZA commercial breeding program was recently simulated in order to explore the potential for implementation of genomic selection (Lin et al. 2016). This process obtained genomic prediction accuracies of 0.19 and 0.71 for biomass yield and heading date, respectively, within the range obtained here from empirical data. The simulation exercise additionally modelled the potential genetic gain from adoption of genomic selection based on the estimated accuracies. A twofold-to-threefold rate of increase was reported, due both to reduced duration of the generation cycle and the ability to select individual spaced plants for sward-based performance (under competition with other plants), the latter being problematic for conventional breeding practice. The observed high degree of concordance between empirical and simulated prediction accuracies for the same breeding program design provides high confidence for beneficial change in genetic gain by twofold-to-threefold through adoption of genomic selection.

The present study has demonstrated the development of a ryegrass reference population based on synthetic population (population genotypes) rather than individual genotypes. The genotypic data are not restricted to specific individual plants in the original trials, but instead relates to the synthetic population, that can be trialled over additional environments. Consequently, multi-environment phenotypic performance data can be gathered and combined with the original genotypic data to develop genomic prediction equations for a broad range of environments.

The outcomes of this study have validated the use of historical data for rapid implementation of genomic selection in existing breeding programs, rather than de novo design and assembly of reference and selection populations, so permitting substantial savings in terms of time and resource costs. However, this approach should be seen not as an alternative, but as complementary, to de novo design programs, which offer the benefits of a broader capture of judiciously selected initial germplasm, capacity to evaluate both individual plants and sward-based populations and the opportunity to implement more accurate and sensitive methods for phenotypic assessment, particularly those based on real-time automated in-field sensor technologies (Badenhorst et al. 2017).

### Author contribution statement

LWP co-conceptualised the project, performed the data analysis and interpretation and drafted the manuscript. CI generated all of the plant germplasm and phenotypic data. RCB, MCD, PT and VOO performed RNA extractions and genotypic assays. NOIC and HDD co-conceptualised the project, assisted in data interpretation and assisted in drafting of the manuscript. GS and JWF co-conceptualised the project and assisted in drafting of the manuscript.

## Electronic supplementary material

Below is the link to the electronic supplementary material.

Supplementary material 1 (DOCX 14 kb)

Supplementary material 2 (DOCX 20 kb)

## References

[CR1] Annicchiarico P, Nazzicari N, Li X, Wei Y, Pecetti L, Brummer EC (2015). Accuracy of genomic selection for alfalfa biomass yield in different reference populations. BMC Genom.

[CR2] Byrne SL, Nagy I, Pfeifer M, Armstead I, Swain S, Studer B, Mayer K, Campbell JD, Czaban A, Hentrup S, Panitz F, Bendixen C, Hedegaard J, Caccamo M, Asp T (2015). A synteny-based draft genome sequence of the forage grass *Lolium perenne*. Plant J.

[CR3] Chapman DF, Cullen BR, Johnson IR, Beca D (2009). Interannual variation in pasture growth rate in Australian and New Zealand dairy regions and its consequences for system management. Anim Prod Sci.

[CR4] Chapman DF, Bryant JR, McMillan WH, Khaembah EN (2012). Economic values for evaluating pasture plant traits. Proc N Z Grassl Assoc.

[CR5] Badenhorst PE, Phelan A, Pembleton LW, Cogan, NOI, Spangenberg GC (2017) Yield assessment of a 270000 plant perennial ryegrass field trial using multispectral aerial imaging platform. In: Proceedings from the UAS4RS 2017 (unmanned aircraft systems for remote sensing) conference, Hobart, Tasmania

[CR6] de Klein CAM (2001). An analysis of environmental and economic implications of nil and restricted grazing systems designed to reduce nitrate leaching from New Zealand dairy farms. II. Pasture production and cost/benefit analysis. N Z J Agric Res.

[CR7] Farrell JD, Byrne S, Paina C, Asp T (2014). De novo assembly of the perennial ryegrass transcriptome using an RNA-Seq strategy. PLoS ONE.

[CR8] Fè D, Cericola F, Byrne S, Lenk I, Ashraf BH, Pedersen MG, Roulund N, Asp T, Janss L, Jensen CS, Jensen J (2015). Genomic dissection and prediction of heading date in perennial ryegrass. BMC Genom.

[CR9] Fè D, Ashraf BH, Pedersen MG, Janss L, Byrne S, Roulund N, Lenk I, Didion T, Asp T, Jensen CS, Jensen J (2016). Accuracy of genomic prediction in a commercial perennial ryegrass breeding program. Plant Genome.

[CR10] Forster JW, Cogan NOI, Dobrowolski MP, van Zijll de Jong E, Spangenberg GC, Smith KF, Kole C, Abbott A (2008). Molecular breeding technologies for forage and turf plants. Principles and practices of plant genomics. Volume 2: molecular breeding.

[CR11] Gilmour AR, Gogel BJ, Cullis BR, Thompson R (2009). ASReml user guide release 3.0.

[CR12] Grinberg NF, Lovatt A, Hegarty M, Lovatt A, Skot KP, Kelly R, Blackmore T, Thorogood D, King RD, Armstead I, Powell W, Skot L (2016). Implementation of genomic prediction in *Lolium perenne* (L.) breeding populations. Front Plant Sci.

[CR13] Hayes BJ, Cogan NOI, Pembleton LW, Goddard ME, Wang J, Spangenberg GC, Forster JW (2013). Prospects for genomic selection in forage plant species. Plant Breed.

[CR14] Hennig C (2015) fpc: flexible procedures for clustering. In: R package version 2.1-10 edn

[CR15] Muranty H, Troggio M (2015). Accuracy and responses of genomic selection on key traits in apple breeding. Hortic Res.

[CR16] Li H, Durbin R (2009). Fast and accurate short read alignment with Burrows–Wheeler transform. Bioinformatics.

[CR17] Li H, Handsaker B (2009). The sequence alignment/map format and SAMtools. Bioinformatics.

[CR18] Li L, Long Y, Zhang L, Dalton-Morgan J, Batley J, Yu L, Meng J, Li M (2015). Genome wide analysis of flowering time trait in multiple environments via high-throughput genotyping technique in *Brassica napus* L. PLoS ONE.

[CR19] Lin Z, Cogan NOI, Pembleton LW, Spangenberg GC, Forster JW, Hayes BJ, Daetwyler HD (2016). Genetic gain and inbreeding from genomic selection in a simulated commercial breeding program for perennial ryegrass. Plant Genome.

[CR20] Malmberg MM, Pembleton LW, Baillie RC, Drayton MC, Sudheesh S, Kaur S, Shinozuka H, Verma P, Spangenberg GC, Daetwyler HD, Forster JW, Cogan NOI (2017). Genotyping-by-sequencing through transcriptomics: implementation in a range of crop species with varying breeding habits and ploidy. Plant Biotechnol J.

[CR21] Meuwissen THE, Hayes BJ, Goddard ME (2001). Prediction of total genetic value using genome-wide dense marker maps. Genetics.

[CR22] Money D, Gardner K, Migicovsky Z, Schwaninger H, Zhong G-Y, Myles S (2015). LinkImpute: fast and accurate genotype imputation for nonmodel organisms. G3 Genes Genome Genet.

[CR23] Pembleton LW, Drayton MC, Bain M, Baillie RC, Inch C, Spangenberg GC, Wang J, Forster JW, Cogan NOI (2016). Targeted genotyping-by-sequencing permits cost-effective identification and discrimination of pasture grass species and cultivars. Theor Appl Genet.

[CR24] Pérez P, de los Campos G (2014). Genome-wide regression and prediction with the BGLR statistical package. Genetics.

[CR25] Rawnsley RP, Chapman DF, Jacobs JL, Garcia SC, Callow MN, Edwards GR, Pembleton KP (2013). Complementary forages—integration at a whole-farm level. Anim Prod Sci.

[CR26] Sampoux J-P, Baudouin P, Bayle B, Beguier V, Bourdon P, Chosson J, Deneufbourg F, Galbrun C, Ghesquiere M, Noel D, Pietraszek W, Tharel B, Viguie A (2011). Breeding perennial grasses for forage usage: an experimental assessment of trait changes in diploid perennial ryegrass (*Lolium perenne* L.) cultivars released in the last four decades. Field Crops Res.

[CR27] Sawbridge T, Ong EK, Binnion C, Emmerling M, McInnes R, Meath K, Nguyen N, Nunan K, O’Neill M, O’Toole F, Rhodes C, Simmonds J, Tian P, Wearne K, Webster T, Winkworth A, Spangenberg G (2003). Generation and analysis of expressed sequence tags in perennial ryegrass (*Lolium perenne* L.). Plant Sci.

[CR28] Shinozuka H, Cogan NOI, Spangenberg GC, Forster JW (2012). Quantitative trait locus (QTL) meta-analysis and comparative genomics for candidate gene prediction in perennial ryegrass (*Lolium perenne* L.). BMC Genet.

[CR29] Shinozuka H, Cogan NOI, Spangenberg GC, Forster JW (2017). Reference transcriptome assembly and annotation for perennial ryegrass. Genome.

[CR30] Smith A, Cullis B, Gilmour A (2001). Applications: the analysis of crop variety evaluation data in Australia. Aust N Z J Stat.

[CR31] Stewart A, Hayes R (2011). Ryegrass breeding—balancing trait priorities. Ir J Agric Food Res.

[CR32] Tayeh N, Klein A, Le Paslier M-C, Jacquin F, Houtin H, Rond C, Chabert-Martinello M, Magnin-Robert J-B, Marget P, Aubert G, Burstin J (2015). Genomic prediction in pea: effect of marker density and training population size and composition on prediction accuracy. Front Plant Sci.

[CR33] Wang J, Cogan NOI, Forster JW (2016). Prospects for applications of genomic tools in registration testing and seed certification of ryegrass varieties. Plant Breed.

[CR34] Wang J, Pembleton LW, Cogan NOI, Forster JW (2016). Evidence for heterosis in italian ryegrass (*Lolium multiflorum* Lam.) based on inbreeding depression in F_2_ generation offspring from biparental crosses. Agronomy.

[CR35] Wilkins PW, Humphreys MO (2003). Progress in breeding perennial forage grasses for temperate agriculture. J Agric Sci.

[CR36] Woodfield DR (1999). Genetic improvements in New Zealand forage cultivars. Proc N Z Grassl Assoc.

[CR37] Yang J, Benyamin B, McEvoy BP, Gordon S, Henders AK, Nyholt DR, Madden PA, Heath AC, Martin NG, Montgomery GW, Goddard ME, Visscher PM (2010). Common SNPs explain a large proportion of the heritability for human height. Nat Genet.

