# Supplementary material for: Exploitation of data from breeding programs supports rapid implementation of genomic selection for key agronomic traits in perennial ryegrass
Source: Theor Appl Genet. 2018 Jun 2;131(9):1891–902. doi: 10.1007/s00122-018-3121-7 (PMC6096624; doi:10.1007/s00122-018-3121-7)
Supplement: Supplementary file 1 — Supplementary material 1 (DOCX 14 kb) [file 122_2018_3121_MOESM1_ESM.docx]

Online Resource 1

**Online Resource 1:** Broad-sense heritability of seasonal biomass yield and heading date. Heritabilites for biomass yield are an average across two trials within each year.

|  | **Broad Sense Heritability (H^2^)** | | | | | | |
| --- | --- | --- | --- | --- | --- | --- | --- |
| **Year** | **Autumn** | **Winter** | **Early Spring** | **Late Spring** | **Summer** | **Average Seasonal** | **Heading Date** |
| 1997 | 0.49 | 0.77 | 0.60 | 0.45 | 0.35 | 0.60 |  |
| 1998 | 0.40 | 0.60 | 0.53 | 0.46 | 0.14 | 0.35 |  |
| 1999 | 0.33 | 0.41 | 0.68 | 0.17 | 0.24 | 0.12 |  |
| 2000 | 0.49 | 0.79 | 0.57 | 0.52 | 0.27 | 0.37 |  |
| 2001 | 0.28 | 0.72 | 0.65 | 0.40 | 0.31 | 0.32 |  |
| 2002 | 0.59 | 0.74 | 0.81 | 0.59 | 0.25 | 0.34 | 0.91 |
| 2003 | 0.30 | 0.39 | 0.62 | 0.40 | 0.28 | 0.26 | 0.86 |
| 2004 | 0.14 | 0.51 | 0.45 | 0.48 | 0.25 | 0.22 | 0.86 |
| 2005 | 0.66 | 0.72 | 0.24 | 0.24 | 0.35 | 0.30 | 0.91 |
| 2006 | 0.28 | 0.67 | 0.16 | 0.25 | 0.22 | 0.33 | 0.65 |
| 2007 | 0.60 | 0.39 | 0.35 | 0.41 | 0.36 | 0.21 | 0.85 |
| 2008 | 0.76 | 0.75 | 0.77 | 0.49 | 0.19 | 0.23 | 0.91 |
| 2009 | 0.59 | 0.65 | 0.70 | 0.51 | 0.24 | 0.40 | 0.93 |
| 2010 | 0.52 | 0.44 | 0.78 | 0.65 | 0.41 | 0.51 | 0.85 |
| 2011 | 0.75 | 0.24 | 0.55 | 0.57 | 0.34 | 0.39 | 0.85 |
| 2012 | 0.23 | 0.49 | 0.41 | 0.13 | 0.05 | 0.19 | 0.92 |
| 2013 | 0.08 | 0.33 | 0.45 | 0.28 | 0.25 | 0.12 |  |
| 2014 | 0.19 | 0.30 | 0.62 | 0.45 | 0.19 | 0.13 |  |
| mean | 0.43 | 0.55 | 0.55 | 0.41 | 0.26 | 0.30 | 0.86 |
