# Supplementary material for: Exploitation of data from breeding programs supports rapid implementation of genomic selection for key agronomic traits in perennial ryegrass
Source: Theor Appl Genet. 2018 Jun 2;131(9):1891–902. doi: 10.1007/s00122-018-3121-7 (PMC6096624; doi:10.1007/s00122-018-3121-7)
Supplement: Supplementary file 2 — Supplementary material 2 (DOCX 20 kb) [file 122_2018_3121_MOESM2_ESM.docx]

Online Resource 2

**Online Resource 2:** Global and Group A genomic prediction accuracy with 1 (Standard), 2 or 3 years between the reference and prediction populations.

|  |  | **Global Prediction Accuracy** | | |
| --- | --- | --- | --- | --- |
| **Year** | **Season** | **1 Year Gap** | **2 Year Gap** | **3 Year Gap** |
| 2004 | Autumn | 0.266 | 0.415 | 0.271 |
| 2005 | Autumn | 0.211 | 0.004 | 0.265 |
| 2006 | Autumn | 0.118 | 0.173 | 0.117 |
| 2007 | Autumn | 0.485 | 0.525 | 0.554 |
| 2008 | Autumn | -0.007 | 0.198 | 0.146 |
| 2009 | Autumn | 0.416 | 0.421 | 0.425 |
| 2010 | Autumn | 0.339 | 0.495 | 0.519 |
| 2011 | Autumn | -0.049 | -0.041 | -0.079 |
| 2012 | Autumn | 0.051 | -0.012 | -0.016 |
| 2013 | Autumn | -0.100 | -0.178 | -0.166 |
| 2014 | Autumn | 0.259 | 0.222 | 0.249 |
| **Mean** | **Autumn** | **0.181** | **0.202** | **0.208** |
| 2004 | Winter | 0.292 | 0.261 | 0.140 |
| 2005 | Winter | 0.018 | 0.075 | -0.084 |
| 2006 | Winter | 0.167 | 0.094 | 0.129 |
| 2007 | Winter | 0.292 | 0.231 | 0.196 |
| 2008 | Winter | 0.425 | 0.417 | 0.398 |
| 2009 | Winter | 0.200 | 0.268 | 0.248 |
| 2010 | Winter | 0.114 | 0.069 | 0.082 |
| 2011 | Winter | 0.053 | 0.020 | -0.011 |
| 2012 | Winter | 0.054 | 0.099 | 0.117 |
| 2013 | Winter | 0.271 | 0.250 | 0.283 |
| 2014 | Winter | 0.270 | 0.229 | 0.212 |
| **Mean** | **Winter** | **0.196** | **0.183** | **0.155** |
| 2004 | Early Spring | 0.588 | 0.541 | 0.419 |
| 2005 | Early Spring | 0.582 | 0.614 | 0.540 |
| 2006 | Early Spring | 0.011 | -0.023 | -0.011 |
| 2007 | Early Spring | 0.576 | 0.577 | 0.571 |
| 2008 | Early Spring | 0.726 | 0.678 | 0.691 |
| 2009 | Early Spring | 0.492 | 0.502 | 0.518 |
| 2010 | Early Spring | 0.659 | 0.669 | 0.698 |
| 2011 | Early Spring | 0.667 | 0.652 | 0.600 |
| 2012 | Early Spring | 0.682 | 0.690 | 0.684 |
| 2013 | Early Spring | 0.514 | 0.514 | 0.493 |
| 2014 | Early Spring | 0.661 | 0.637 | 0.646 |
| **Mean** | **Early Spring** | **0.560** | **0.550** | **0.532** |
| 2004 | Late Spring | 0.466 | 0.012 | 0.182 |
| 2005 | Late Spring | 0.273 | 0.254 | 0.112 |
| 2006 | Late Spring | 0.220 | 0.164 | 0.165 |
| 2007 | Late Spring | 0.149 | 0.216 | 0.293 |
| 2008 | Late Spring | 0.108 | 0.163 | 0.202 |
| 2009 | Late Spring | 0.532 | 0.533 | 0.483 |
| 2010 | Late Spring | 0.206 | 0.218 | 0.156 |
| 2011 | Late Spring | 0.515 | 0.514 | 0.447 |
| 2012 | Late Spring | 0.137 | 0.112 | 0.106 |
| 2013 | Late Spring | 0.346 | 0.369 | 0.317 |
| 2014 | Late Spring | 0.222 | 0.183 | 0.146 |
| **Mean** | **Late Spring** | **0.289** | **0.249** | **0.237** |
| 2004 | Summer | 0.431 | 0.457 | 0.430 |
| 2005 | Summer | -0.104 | -0.224 | -0.193 |
| 2006 | Summer | 0.280 | 0.237 | 0.199 |
| 2007 | Summer | 0.374 | 0.350 | 0.389 |
| 2008 | Summer | 0.003 | 0.036 | 0.029 |
| 2009 | Summer | 0.309 | 0.319 | 0.265 |
| 2010 | Summer | 0.405 | 0.430 | 0.422 |
| 2011 | Summer | 0.472 | 0.483 | 0.498 |
| 2012 | Summer | -0.071 | -0.114 | -0.205 |
| 2013 | Summer | 0.381 | 0.370 | 0.371 |
| 2014 | Summer | 0.178 | 0.401 | 0.319 |
| **Mean** | **Summer** | **0.242** | **0.249** | **0.229** |
| 2004 | Average | 0.246 | 0.258 | 0.364 |
| 2005 | Average | 0.035 | 0.000 | 0.033 |
| 2006 | Average | 0.159 | 0.099 | 0.098 |
| 2007 | Average | 0.352 | 0.382 | 0.352 |
| 2008 | Average | 0.214 | 0.212 | 0.255 |
| 2009 | Average | 0.363 | 0.394 | 0.375 |
| 2010 | Average | 0.575 | 0.539 | 0.536 |
| 2011 | Average | 0.159 | 0.193 | 0.227 |
| 2012 | Average | 0.423 | 0.368 | 0.336 |
| 2013 | Average | 0.496 | 0.508 | 0.463 |
| 2014 | Average | 0.155 | 0.141 | 0.152 |
| **Mean** | **Average** | **0.289** | **0.281** | **0.290** |

|  |  | **Group A Prediction Accuracy** | | |
| --- | --- | --- | --- | --- |
| **Year** | **Season** | **1 Year Gap** | **2 Year Gap** | **3 Year Gap** |
| 2004 | Autumn | 0.327 | 0.389 | 0.467 |
| 2005 | Autumn | 0.317 | 0.193 | 0.331 |
| 2006 | Autumn | 0.530 | 0.534 | 0.501 |
| 2007 | Autumn | 0.631 | 0.531 | 0.532 |
| 2008 | Autumn | 0.201 | 0.288 | 0.171 |
| 2009 | Autumn | 0.314 | 0.313 | 0.379 |
| 2010 | Autumn | 0.390 | 0.468 | 0.503 |
| 2011 | Autumn | -0.036 | -0.076 | -0.060 |
| 2012 | Autumn | 0.200 | 0.132 | 0.078 |
| 2013 | Autumn | -0.159 | -0.266 | -0.251 |
| 2014 | Autumn | 0.513 | 0.479 | 0.496 |
| **Mean** | **Autumn** | **0.294** | **0.271** | **0.286** |
| 2004 | Winter | 0.153 | 0.118 | 0.528 |
| 2005 | Winter | 0.128 | 0.066 | 0.014 |
| 2006 | Winter | 0.355 | 0.302 | 0.301 |
| 2007 | Winter | 0.598 | 0.491 | 0.416 |
| 2008 | Winter | 0.424 | 0.427 | 0.461 |
| 2009 | Winter | 0.269 | 0.295 | 0.255 |
| 2010 | Winter | -0.026 | -0.068 | -0.044 |
| 2011 | Winter | -0.008 | -0.028 | -0.058 |
| 2012 | Winter | 0.125 | 0.185 | 0.231 |
| 2013 | Winter | 0.139 | 0.155 | 0.150 |
| 2014 | Winter | 0.197 | 0.189 | 0.180 |
| **Mean** | **Winter** | **0.214** | **0.194** | **0.221** |
| 2004 | Early Spring | 0.655 | 0.638 | -0.049 |
| 2005 | Early Spring | 0.684 | 0.672 | 0.670 |
| 2006 | Early Spring | 0.109 | 0.057 | 0.095 |
| 2007 | Early Spring | 0.806 | 0.785 | 0.794 |
| 2008 | Early Spring | 0.759 | 0.737 | 0.774 |
| 2009 | Early Spring | 0.534 | 0.515 | 0.515 |
| 2010 | Early Spring | 0.646 | 0.678 | 0.701 |
| 2011 | Early Spring | 0.651 | 0.651 | 0.613 |
| 2012 | Early Spring | 0.594 | 0.590 | 0.587 |
| 2013 | Early Spring | 0.456 | 0.481 | 0.472 |
| 2014 | Early Spring | 0.718 | 0.716 | 0.722 |
| **Mean** | **Early Spring** | **0.601** | **0.593** | **0.536** |
| 2004 | Late Spring | 0.589 | 0.298 | 0.246 |
| 2005 | Late Spring | 0.329 | 0.302 | 0.143 |
| 2006 | Late Spring | 0.296 | 0.247 | 0.201 |
| 2007 | Late Spring | 0.345 | 0.270 | 0.305 |
| 2008 | Late Spring | 0.250 | 0.333 | 0.292 |
| 2009 | Late Spring | 0.392 | 0.456 | 0.427 |
| 2010 | Late Spring | 0.164 | 0.138 | 0.128 |
| 2011 | Late Spring | 0.461 | 0.470 | 0.395 |
| 2012 | Late Spring | 0.058 | 0.069 | 0.049 |
| 2013 | Late Spring | 0.438 | 0.469 | 0.393 |
| 2014 | Late Spring | 0.469 | 0.245 | 0.103 |
| **Mean** | **Late Spring** | **0.345** | **0.300** | **0.244** |
| 2004 | Summer | 0.376 | 0.389 | 0.075 |
| 2005 | Summer | -0.017 | -0.082 | -0.069 |
| 2006 | Summer | 0.548 | 0.493 | 0.465 |
| 2007 | Summer | 0.318 | 0.289 | 0.293 |
| 2008 | Summer | -0.060 | 0.030 | -0.009 |
| 2009 | Summer | 0.371 | 0.372 | 0.375 |
| 2010 | Summer | 0.402 | 0.396 | 0.393 |
| 2011 | Summer | 0.300 | 0.306 | 0.324 |
| 2012 | Summer | -0.041 | -0.070 | -0.173 |
| 2013 | Summer | 0.234 | 0.181 | 0.189 |
| 2014 | Summer | 0.442 | 0.304 | 0.329 |
| **Mean** | **Summer** | **0.261** | **0.237** | **0.199** |
| 2004 | Average | 0.241 | 0.290 | 0.337 |
| 2005 | Average | 0.304 | 0.195 | 0.250 |
| 2006 | Average | 0.218 | 0.123 | 0.111 |
| 2007 | Average | 0.592 | 0.556 | 0.475 |
| 2008 | Average | 0.035 | 0.209 | 0.321 |
| 2009 | Average | 0.393 | 0.408 | 0.387 |
| 2010 | Average | 0.554 | 0.520 | 0.510 |
| 2011 | Average | 0.170 | 0.189 | 0.225 |
| 2012 | Average | 0.349 | 0.311 | 0.305 |
| 2013 | Average | 0.565 | 0.564 | 0.573 |
| 2014 | Average | 0.521 | 0.425 | 0.386 |
| **Mean** | **Average** | **0.358** | **0.344** | **0.353** |
